# From Molecules to Behavior in Long-Term Inorganic Mercury Intoxication: Unraveling Proteomic Features in Cerebellar Neurodegeneration of Rats

**DOI:** 10.3390/ijms23010111

**Published:** 2021-12-22

**Authors:** Leonardo Oliveira Bittencourt, Victória Santos Chemelo, Walessa Alana Bragança Aragão, Bruna Puty, Aline Dionizio, Francisco Bruno Teixeira, Mileni Silva Fernandes, Márcia Cristina Freitas Silva, Luanna Melo Pereira Fernandes, Edivaldo Herculano Corrêa de Oliveira, Marilia Afonso Rabelo Buzalaf, Maria Elena Crespo-Lopez, Cristiane do Socorro Ferraz Maia, Rafael Rodrigues Lima

**Affiliations:** 1 Laboratory of Functional and Structural Biology, Institute of Biological Sciences, Federal University of Pará, Belém 66075-110, PA, Brazil; leo.bittencourt25@gmail.com (L.O.B.); vicchemelo@gmail.com (V.S.C.); walessa.aragao@gmail.com (W.A.B.A.); brunaputy@gmail.com (B.P.); teixeira.f.bruno@gmail.com (F.B.T.); marciaf@ufpa.br (M.C.F.S.); 2 Department of Biological Sciences, Bauru Dental School, University of São Paulo, Bauru 17012-901, SP, Brazil; alinesdionizio@usp.br (A.D.); mi_biol@yahoo.com.br (M.S.F.); mbuzalaf@fob.usp.br (M.A.R.B.); 3 Laboratory Pharmacology of Inflammation and Behavior, Institute of Health Sciences, Federal University of Pará, Belém 66075-110, PA, Brazil; luannafe@hotmail.com (L.M.P.F.); crismaia@ufpa.br (C.d.S.F.M.); 4 Laboratory of Cytogenetics, Environmental Session, Evandro Chagas Institute, Ananindeua 66093-020, PA, Brazil; ehco@ufpa.br; 5 Laboratory of Molecular Pharmacology, Institute of Biological Sciences, Federal University of Pará, Belém 66075-110, PA, Brazil; maria.elena.crespo.lopez@gmail.com

**Keywords:** mercury, central nervous system, proteomic, motor functions

## Abstract

Mercury is a severe environmental pollutant with neurotoxic effects, especially when exposed for long periods. Although there are several evidences regarding mercury toxicity, little is known about inorganic mercury (IHg) species and cerebellum, one of the main targets of mercury associated with the neurological symptomatology of mercurial poisoning. Besides that, the global proteomic profile assessment is a valuable tool to screen possible biomarkers and elucidate molecular targets of mercury neurotoxicity; however, the literature is still scarce. Thus, this study aimed to investigate the effects of long-term exposure to IHg in adult rats’ cerebellum and explore the modulation of the cerebellar proteome associated with biochemical and functional outcomes, providing evidence, in a translational perspective, of new mercury toxicity targets and possible biomarkers. Fifty-four adult rats were exposed to 0.375 mg/kg of HgCl_2_ or distilled water for 45 days using intragastric gavage. Then, the motor functions were evaluated by rotarod and inclined plane. The cerebellum was collected to quantify mercury levels, to assess the antioxidant activity against peroxyl radicals (ACAPs), the lipid peroxidation (LPO), the proteomic profile, the cell death nature by cytotoxicity and apoptosis, and the Purkinje cells density. The IHg exposure increased mercury levels in the cerebellum, reducing ACAP and increasing LPO. The proteomic approach revealed a total 419 proteins with different statuses of regulation, associated with different biological processes, such as synaptic signaling, energy metabolism and nervous system development, e.g., all these molecular changes are associated with increased cytotoxicity and apoptosis, with a neurodegenerative pattern on Purkinje cells layer and poor motor coordination and balance. In conclusion, all these findings feature a neurodegenerative process triggered by IHg in the cerebellum that culminated into motor functions deficits, which are associated with several molecular features and may be related to the clinical outcomes of people exposed to the toxicant.

## 1. Introduction

Mercury is a hazardous toxic pollutant distributed in the environment and is considered a severe public health concern [1,2]. Humans are subjected to the mercurial compound by different sources because of anthropogenic activities, such as occupational exposure and environmental contamination by illegal gold mining that contaminates fish and seafood [3,4]. Elemental mercury (Hg^0^) and methylmercury (MeHg) are considered the main species of exposure in occupational and environmental (via seafood) outbreaks, respectively [3,4]. However, even in the case of MeHg exposure, consequences would additionally be due to inorganic mercury (IHg), since the latter specie was detected in both contaminated food and cells of central nervous system (CNS) origin [5,6].

Considering the presence of IHg in fish, this becomes a serious public health problem since those who consume them, especially populations whose food bases are these items [7], are subject to prolonged exposure. Some evidence points out that commercially available predatory species of fish have significant levels of IHg, reaching mean values of 0.15 µg/g of IHg [6]. Moreover, although IHg species present lower liposolubility than organic ones, they also cause damage. Due to its toxicokinetic characteristics, after absorption, kidneys [8] and the blood and cells from the human central nervous system (CNS) are also affected by IHg poisoning [9,10,11].

Neurological manifestations are the primary symptomatology of chronic mercury poisoning, including cognitive impairments, tremors, and ataxia [4]. Tremors and ataxia are symptoms associated with loss of motor control and higher brain function [12], and when it comes to motor functions, the cerebellum is a pivotal structure of the CNS that plays an essential role in cognitive and motor skills [12,13]. The organ is a dense network of reciprocal crossed cerebellum-cerebral connections, interconnected with supratentorial motor areas, paralimbic, and associated with cortices pathways [12,13]. Moreover, damages in cerebellum interrupt the complex cerebellar circuitry between the neurons of the cerebellar cortex which brings clinical implications, including multiple system atrophy, spinocerebellar ataxia, hypoxia in Purkinje cells, associated with neuropathologies, such as autism spectrum disorder, attention deficit-hyperactivity disorder, and developmental dyslexia [14,15].

In this way, seeking to investigate even more the molecular features associated with IHg-induced neurotoxicity, our group has been researching the effects of IHg exposure in rats under a model that has shown to be capable of generating outcomes similar to those observed in humans, from systemic to regional analyses [16,17,18,19,20,21]. Moreover, some of these studies showed damages to essential areas to motor function, such as the motor cortex [19,20,21] and spinal cord [18]. However, the relationship between long-term exposure to IHg and its influence on the biochemistry and functionality of the cerebellum still lacks evidence. Thus, this study aimed to investigate the global proteomic profile underlying IHg-induced neurotoxicity associated with the motor dysfunction outcome, including molecular, biochemical, and morphological approaches.

## 2. Results

### 2.1. Long-Term Exposure to IHg Did Not Impair Mass Body Gain of Adult Rats

**Figure 1:**
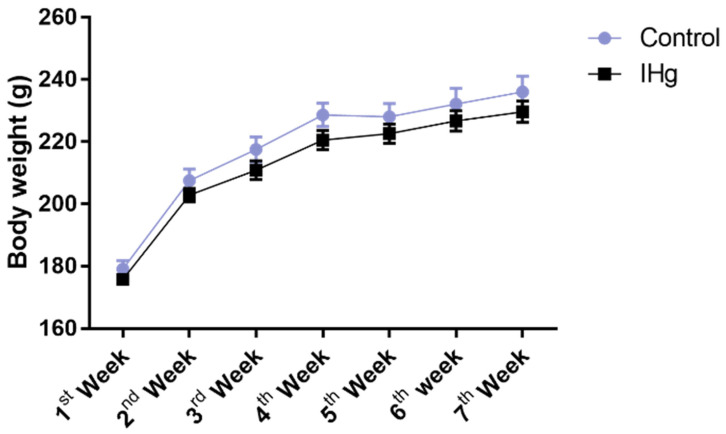
Body mass (g) register of adult rats exposed to 0.375 mg/kg of HgCl_2_ for 45 days. Results are expressed as mean ± S.E.M. Two-way ANOVA with Sidak‘s post-test, *p* > 0.05.

The exposure to IHg did not cause any body weight change over time (*p* > 0.05), and no loss of animals was observed (Figure 1).

### 2.2. Prolonged IHg Exposure Increased the Total Mercury Content in Cerebellar Parenchyma of Adult Rats

**Figure 2:**
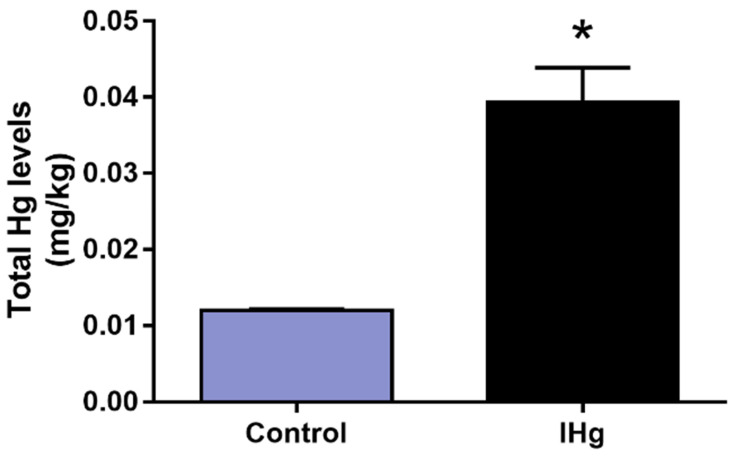
Total mercury (Hg) content in the cerebellum of adult rats exposed to 0.375 mg/kg of HgCl_2_ for 45 days. Results are expressed as mg/kg (mean ± S.E.M.), * *p* < 0.05, *t*-Student test (n = 10).

The total mercury levels were significantly higher (*p* = 0.0003) in the IHg group (0.0394 ± 0.004 mg/kg) in comparison with the control group (0.0120 ± 0.0001703 mg/kg), as observed in Figure 2.

### 2.3. IHg Long-Term Exposure Triggered Oxidative Stress in the Cerebellum of Adult Rats

**Figure 3:**
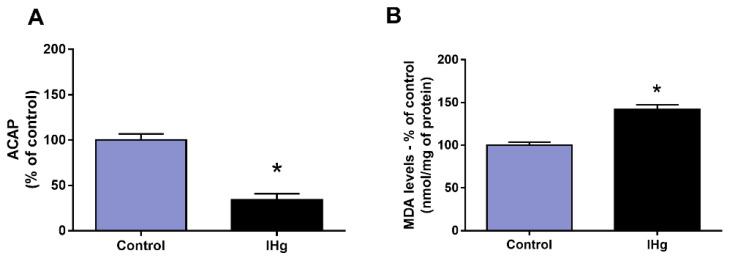
Oxidative biochemistry analyses of adult rats’ cerebellum exposed to 0.375 mg/kg of HgCl_2_ for 45 days. In (**A**) analyses of Antioxidant Capacity Against Peroxyl Radicals (ACAPs) and (**B**) lipid peroxidation (LPO). Data presented as percentage (%) of control (mean ± S.E.M.). * *p* < 0.05, *t*-Student test (n = 10).

The IHg reduced the cerebellar ACAP in 66.15% (±7%) in comparison with the control group (100 ± 6.67%; *p* = 0.0002) and in parallel, the LPO increased 41.70% (±5.75) when compared with the control (100 ± 3.63%) (*p* = 0.023) (Figure 3).

### 2.4. The Cerebellar Proteomic Profile Was Significantly Modulated after Exposure to IHg Rats

The proteomic approach revealed the modulation of 419 proteins in the cerebellum of rats exposed to IHg. Among them, 24 proteins with exclusive expression in the IHg-exposed group and 166 with unique expression in the control group. Moreover, 196 proteins were found down-regulated and 33 up-regulated. Table 1 shows some proteins highlighted in the discussion, and the complete data are available in Supplementary Table S1 (See Supplementary Materials).

**Figure 4:**
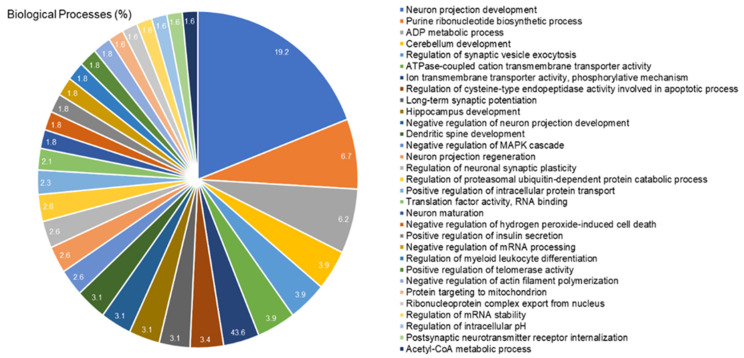
Functional distribution of proteins identified with differential expression in the cerebellum of rats exposed to IHg vs. control group. Categories of proteins based on gene ontology annotation of biological process. Terms significant (Kappa Score =  0.4) and distribution according to the percentage of the number of genes. UNIPROT provided proteins access number. The gene ontology was evaluated according to the ClueGo^®^ plugin of Cytoscape^®^ software 3.7.

The bioinformatic analysis showed 31 biological processes associated with the proteins modulated in the cerebellum of exposed rats (Figure 4). Among them, the five most impaired processes were neuron projection development (19.2%), purine ribonucleotide biosynthetic process (6.7%), ADP metabolic process (6.2%), cerebellum development (3.9%), and regulation of synaptic vesicle exocytosis (3.9%).

### 2.5. The IHg Exposure Increased Cell Death by Cytotoxicity and Apoptosis in the Cerebellum of Adult Rats

**Figure 5:**
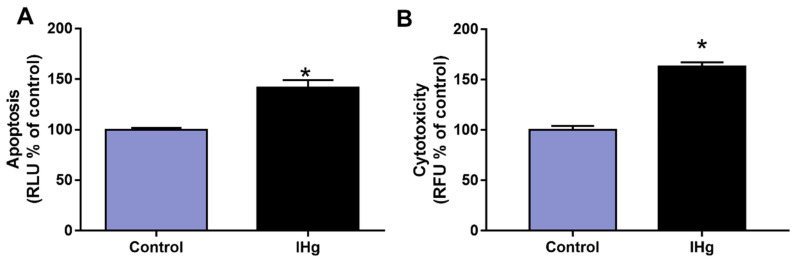
Nature of cell death analyses in the cerebellum of adult rats exposed to 0.375 mg/kg of HgCl_2_ for 45 days. The apoptosis analysis (**A**) was initially represented as relative luminescence unit (RLU), and cytotoxicity analysis (**B**) was expressed initially as relative fluorescence unit (RFU). Data are presented as percentage (%) of control (mean ± S.E.M.). * *p* < 0.05, *t*-Student test (n = 10).

After 45 days of exposure, the cell death markers of apoptosis increased around 41.70% (±7.35%) in comparison with the control (100 ± 1.81%; *p* = 0.0002), and in parallel, the marker of cytotoxicity increased around 63.10% (±4.19%) in comparison with the control (100 ± 1.66%; *p* < 0.05), as observed in Figure 5.

### 2.6. The IHg Intoxication Reduces Purkinje Cells Density in Adult Rats

**Figure 6:**
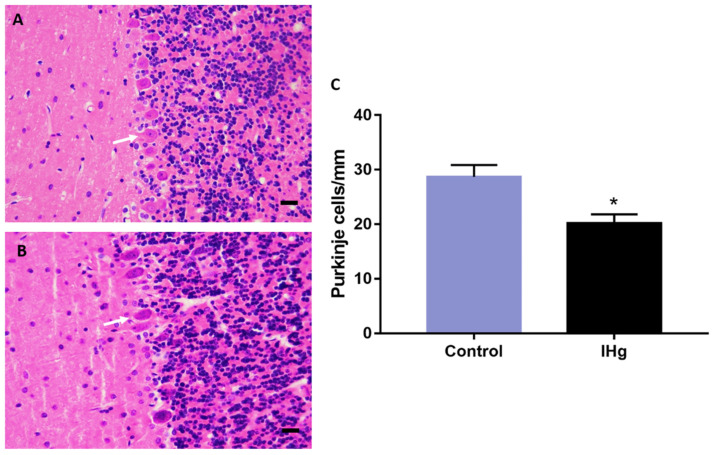
Morphological analysis of the cerebellum of rats exposed to 0.375 mg/kg of HgCl_2_ for 45 days. In (**A**,**B**), representative photomicrographs of control and exposed groups, respectively. In (**C**), results are expressed as mean ± S.E.M and * *p* < 0.05, *t*-Student test (n = 12). Scale bar: 20 µm.

The histological analysis showed a significant reduction in Purkinje cells number in rats (20.22 ± 1.593 cells/mm) in comparison with the control (28.67 ± 2.198 cells/mm; *p* = 0.011) (Figure 6).

### 2.7. The Successive IHg-Induced Damages on the Cerebellum of Rats Led to a Poor Motor Function

**Figure 7:**
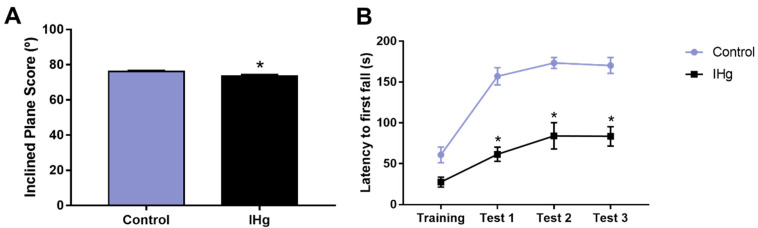
Inclined plane and rotarod performances of rats exposed to 0.375 mg/kg of HgCl_2_ for 45 days. In (**A**), an average of the falling degree of five test sessions in inclined plane. In (**B**), latency (seconds) to the first fall in rotarod. Results are expressed as mean ± S.E.M. * *p* < 0.05, *t*-Student test (**A**) or one-way ANOVA with repeated measures (**B**) (n = 20).

The exposed animals showed a worse motor performance on the inclined plane than the control group (*p* = 0.0129). The control group showed a score of 76.2° (± 0.51°), while the exposed group 73.5° (±0.83°) (Figure 7A). Moreover, the cerebellar functions associated with the rotarod performance were affected by IHg long-term exposure. The latency to the first fall in the exposed group was shorter than the control group in all test sessions (*p* < 0.0001, Figure 7B).

## 3. Discussion

This study is the first investigation that brings to the literature evidence regarding the cerebellar global proteomic profile of rats exposed to IHg that underlies the IHg-induced neurotoxicity. Our results demonstrate the IHg exposure triggers oxidative stress and cell death by cytotoxicity and apoptosis, besides the reduction in Purkinje cells density in the cerebellum of rats. Those changes drove to impairments on cerebellar-related motor function as an inclined-plan and rotarod performance. In addition, the cerebellar proteome of IHg exposed rats revealed the modulation of proteins associated with cerebellar development, proteostasis, synaptic activity, and metabolic functions, which may be underlying the impairments on cerebellar-related motor function of adult rats.

IHg is known for its lower liposolubility, hence the lower capacity of crossing biological barriers as cell membranes, blood-brain-barrier, and placental barrier [22]. The detrimental effects IHg are directly associated with the oxidation state of the ion, i.e., mercurous (Hg^+^) ion causes minor damage in comparison with mercuric ions (Hg^2+^) [22], in which the latter was the chemical form used in this study. Although inorganic species have unfavorable physical-chemical characteristics to biomagnify and bioaccumulate, the presence of IHg in contaminated fish commercially available and consumed by humans was already evidenced [6]. In the past few years, our group has shown that several other organs are susceptible to IHg toxicity, such as blood [16], salivary glands [23], and the central nervous structures such as the hippocampus, motor cortex, and spinal cord [17,18,19,20,21] after the exposure to 0.375 mg/kg per day of HgCl_2_. Then, despite all the toxicokinetic features, the long-term exposure to IHg increased the levels of total mercury in cerebellar parenchyma of adult rats about 4-fold higher than non-exposed animals significantly.

The literature shows that after systemic distribution, IHg can trigger cellular damage such as inhibition of microtubule assembly [24], DNA damage [25,26], mitochondrial impairments [27,28], and oxidative stress [17,29], which in turn, can trigger or aggravate those previously mentioned targets of mercury-induced toxicity. Oxidative stress is caused after an imbalance of cellular redox status, when there is an overproduction of reactive oxygen species (ROS) in detriment to the lower antioxidant capacity [29], being an essential mechanism of mercury damage as previously reviewed [30]. In addition, these biochemical impairments are also visualized in a systemic perspective once we observed oxidative stress triggering in peripheral blood of rats exposed to IHg under the same experimental protocol [16].

To investigate whether this dose of IHg would be capable of reproducing the classic features of mercury mechanisms of damage, we assessed the redox status by the cerebellar capacity of neutralizing peroxyl radicals and the lipid peroxidation. The peroxyl radical affects cells by oxidizing the DNA [31] and initializing the lipid peroxidation of cell and organelle membranes [32]. There is an essential link between peroxyl radicals and lipid peroxidation, which is why we also evaluated the malondialdehyde levels, that indicate the oxidation of polyunsaturated fatty acids present in cell and organelle membranes [33]. Our results showed a decrease in ACAP and increased levels of malondialdehyde, indicating a process of oxidative stress in the cerebellum of rats exposed to IHg, which is consistent with the literature on mercury toxicity, and possibly associated with the down-regulation of Peroxiredoxin 2 (P35704) and 6 (O35244), and Superoxide dismutase (P07895) as shown in the proteomics, suggesting that the decrease on enzymatic antioxidant competence against ROS, such as superoxide anions, peroxides and peroxide nitrites derived from IHg-induced toxicity increased lipid peroxidation [34,35].

Oxidative stress impairs several biological processes, such as mitochondrial function, energy metabolism, cell death, synaptic plasticity, and protein homeostasis (proteostasis) [36,37,38,39]. As part of the energy imbalance, the reduction in ATP levels may lead to a transient downregulation of global translation processes to generate new proteins, and, indeed, in time, an oxidative stress state; this is a natural strategy to prevent protein unfolding and aggregation [40,41]. Corroborating this fact, the proteomic approach showed a significant reduction in translation processes in the cerebellum of rats exposed to IHg, as shown by the 362 proteins down-regulated or exclusively found in the control group.

In addition, the adequate regulation of the ubiquitin-proteasome and molecular chaperones, such as heat shock proteins (HSP), is involved in proteome protection [40,42]. The former is a proteolytic system that degrades oxidized proteins, working as quality control, and the latter, mainly the HSP, is related to protein maturation, re-folding, and degradation [41,43]. The proteomic approach showed the down-regulation of Ubiquitin-like modifier-activating enzyme 1 (Q5U300) and exclusive regulation of E3 ubiquitin-protein ligase UBR5 (Q62671), proteasome subunit alpha type-5 (P34064), small ubiquitin-related modifier 2 and 3 (P61959 and Q5XIF4, respectively), ubiquitin thioesterase OTUB1 (B2RYG6) and ubiquitin-conjugating enzyme E2 variant 2 (Q7M767) in the control group (i.e., absent in exposed group), revealing an evident impairment on the ubiquitin-proteasome system. Furthermore, we also observed the exclusive regulation of HSP 70 kDa protein 4 and 105 kDa (O88600 and Q66HA8, respectively) in the control group and down-regulation of several HSP with different molecular weights as 60 kDa (P63039), 70 kDa (P0DMW0), 90 (P82995). It is worth mentioning that HSP with molecular weights between 40 and 105 kDa are ATP-dependent [44], and interestingly, our proteomic approach also demonstrated the down-regulation of several ATP-synthase subunits (e.g., Q06647, P31399, P10719), suggesting not only a cellular energy metabolism failure but also a compromise on HSP functioning. In fact, HSPs have been previously proposed as important therapeutic tools for many diseases [45], and here we provide evidence on their possible role as early biomarkers of neurological damage.

Besides the oxidation of essential macromolecules as proteins and lipids, we also had the firing of cell death pathways after mitochondrial impairment [46]. In this way, cytochrome c plays a crucial role in apoptosis once its release from mitochondria to cytoplasm promotes the generation of the apoptosome and further activation of pro-caspases 3 and 7 [47]. The proteomic analysis showed the modulation of some cytochrome c oxidase subunits, such as subunits 4 (P10888, up-regulated), 2 (P00406, down-regulated), and 5B (P12075, down-regulated). Corroborating these findings, we also observed increased apoptosis markers (caspase 3/7) in the IHg-exposed group. Regarding the result of cytotoxicity, it ratifies the outcome triggered by apoptotic mechanisms once the method used assesses the activity of a protease released from cells when there is a compromised membrane.

In addition to that, a reduction in Purkinje cell density was observed. These cells play a pivotal role in motor functions, so we elected them to perform the histological analysis. Indeed, the cerebellum presents several structures and different cells; however, the Purkinje cells are integrators and effectors in cerebellar neuronal communication and are important to sensorimotor calibration and motor learning. Each cell receives inputs from several parallel fibers, besides inputs and synaptic contacts from climbing fibers to regulate the movements [48,49,50]. In this way, our data indicate that the increase in apoptosis and cytotoxicity markers, besides the oxidative stress, may reduce Purkinje cell numbers.

Besides the structural component, it is important to highlight the role of proteins related to synaptic communication in neural functions. Firstly, we must clarify that synaptic transmission has a complex dynamic involving the formation, transport, docking of vesicles, the neurotransmitters, the rearrangement of the pre- and post-synaptic neuronal cytoskeleton and the calcium homeostasis [51,52]. Then, the first set of proteins that we observed significantly changed was calmodulin unities 1 (P0DP29), 2 (P0DP30), and 3 (P0DP31) found to be up-regulated, while calcium-calmodulin-dependent protein kinase type II subunit(s) alpha (P11275), beta (P08413), gamma (P11730), and delta (P15791) were down-regulated. The second set of proteins are constituents of the cytoskeleton, such as actin subunits (e.g., P68035 and P60711) down-regulation; microtubule-related proteins, as expected due to the mercury toxicity mechanisms, such as Dynamin 1, 2, and 3 (P21575, P39052, and Q08877, respectively, down-regulated); and several tubulin subunits. Calcium-calmodulin kinase II proteins play several biological roles, such as neurotransmitter synthesis and release, in cognitive and motor functions [53,54], while actin and microtubule-related proteins are important for cytosolic transport vesicles, exocytosis, and endocytosis of vesicles [55].

Comprising the third set of proteins involved in synaptic transmission, we highlight the vesicle constituents, such as synaptotagmin-1 (P21707, up-regulated) and synapsin-1 (P09951, down-regulated). However, four proteins must be emphasized: synaptophysin (P07825; up-regulated); microtubule-associated proteins 1A (P34926; up-regulated) and 6 (Q63560; absent in exposed group) and Disks large homolog 4 (P31016, absent in exposed group). These proteins represent one of the most studied synaptic complexes because they are components of synaptic vesicles [56], besides the pre-and post-synaptic platforms [44,57]. This modulation on the synaptosome suggests an impact on cerebellar functions caused by IHg long-term exposure, which may have led to the loss of balance and motor control demonstrated by the behavioral assessment.

For cerebellar functions and development, structural proteins play a crucial role in the action potential and synaptic communication. Considering that, we observed the down-regulation of myelin basic protein (P02688), the up-regulation of myelin-oligodendrocyte glycoprotein (Q63345), and exclusive regulation of myelin-associated glycoprotein (P07722) in the control group, indicating damages to the myelin-sheath structure. P02688 is an abundant protein in CNS, and it acts to mediate the cytosolic adhesion of multilayered compact myelin [58]. Besides that, P07722 and Q63345 are proteins associated with the myelin maintenance and axon-glial interplay [59,60]. Thus, modifications on the regulation status of those proteins may compromise the cerebellar functioning by myelin decompaction and axonal regeneration, which impair the action potentials of neurons, and hence, the cerebellum-related motor functions.

In this way, it is evident that IHg triggers considerable damages to the cerebellum, since in the proposed model, the increase in total mercury levels in cerebellar parenchyma was associated with oxidative stress triggering and a neurodegenerative pattern, possibly associated with the cytotoxic and apoptotic mechanism. Besides that, the global proteomic profile revealed several proteins that underlie these successive damages, culminating in the motor dysfunction of rats, as evidenced by rotarod and inclined plane assessment. The motor function depends on several anatomical regions in CNS, and the cerebellum plays a crucial role in balance, motor refinement, and even cognitive aspects [61]. For that reason, we used rotarod and inclined plane tests to assess the rats’ abilities and validate the functional damages triggered by IHg long-term exposure. The results showed an evident impairment in balance and motor refinement as an outcome of IHg exposure and these data reinforce the understanding that IHg affects multiple motor-related regions from CNS.

## 4. Materials and Methods

### 4.1. Ethical Statement and Experimental Protocol

All experiments were performed following bioethical instructions after authorization from the Ethics Committee on Animal Experimentation from the Federal University of Pará under protocol NO 9228050418 and followed the ARRIVE 2.0 guideline (see Supplementary Table S2).

Fifty-four male Wistar rats (*Rattus novergicus*), weighing about 200 g and 90 days old, were used in this study. The animals were divided into two groups (control and exposed) by simple randomization and kept in plastic cages (4 animals per cage). The animals were maintained at a temperature of 25 °C with a 12 h dark/light cycle (lights on at 7 a.m.). The exposed group received a single concentration of 0.375 mg/kg/day for 45 days as HgCl_2_ (Sigma-Aldrich, San Louis, MO, USA) solubilized in distilled water and administered by oral gavage. This dosage was previously established and seen as capable of triggering damage in CNS structures [17,18,19,20,21]; besides, the dose was adjusted weekly according to the body weight. The control group received only distilled water by intragastric gavage for the same period and proportional volume. The animals would be excluded in case of malnutrition and the presence of bruises on the body. During the experimental period there were no deaths and exclusions.

### 4.2. Behavioral Assessment

After 24 h from the last exposure to IHg, behavioral tests were performed. Afterward, ten random animals were conducted to the assay room with attenuation of noise levels and low illumination (12 lux) and acclimated for at least one hour before the behavioral tests to assess the motor functions associated with the cerebellum as primary outcomes. The animals were identified as sequential numbers by L.O.B and V.S.C., and the researchers who performed the behavioral assessment were blinded regarding this information.

#### 4.2.1. Inclined Plane Test

The animals’ ability to maintain its postural stability, a crucial cerebellar skill, was assessed with the inclined plane test. The rats were positioned individually in an apparatus consisting of an inclined plane with adjustable angulation. The angle is raised every 5 degrees (from 0° to 90°) every 5 s, which can be used as an index of climbing strength. The animals were subjected to five test sessions in which the maximum inclination that the animal was able to maintain itself for 5 s was recorded as the final degree. The average angle of the five sessions was analyzed [62,63].

#### 4.2.2. Rotarod Test

To evaluate the motor coordination and balance through a forced task, we performed the rotarod test, consisting of an apparatus (Insight Scientific Equipment, Ribeirão Preto, Brazil) of a grooved metal roll (8 cm in diameter) with separated compartments for each animal. The animals were initially trained to stay on the rotating rod at 15 revolutions per minute (rpm) for 3 min (training phase). Then, we assessed the animal’s ability to remain on the roll for three successive sessions of 3 min each at 15 rpm with an interval of 60 s [64] to evaluate the latency to first fall.

### 4.3. Sample Collection and Preparation

After behavioral assays, the animals were deeply anesthetized with a solution of xylazine hydrochloride (30 mg/kg) and ketamine chloride (180 mg/kg) (i.p.). Then, after total loss of paw and corneal reflexes, the animals were euthanized. The cerebellums were collected after brain removal, and some specimens were gently washed in cold saline solution. After, they were stored in microtubes, frozen in liquid nitrogen, and kept at −80 °C until further procedures, while others were dissociated by collagenase as detailed below, both to assess secondary outcomes.

Before the oxidative biochemistry analyses, the samples were thawed and resuspended (1:1, *w*/*v*) in Tris-HCl buffer (20 mM, pH 7.4, at 4 °C) and sonically disaggregated. Then, each sample was divided into two aliquots and frozen. For mercury measurements and proteomic analysis, the entire samples were frozen until further analyses. The evaluation of apoptosis and cytotoxicity required tissue dissociation right after the sample collection, executed with collagenase in concentrations of 2 mg/mL (20 min) and 4 mg/mL (40 min) at 37 °C. Details are described in the respective topics below.

### 4.4. Mercury Measurements

The samples were digested in a nitric acid, perchloric acid, and sulfuric acid solution (1:1:5). The samples were heated, and the Hg^2+^ present in the samples was converted into Hg^0^ after stannous chloride addition. The equipment used was the Semi-Automatic Mercury Analyzer-Hg 201 (Sanso Seisakusho Co. Ltd., Tokyo, Japan) as previously reproduced by our group [16,65,66,67], following the protocol of Suzuki et al. [68] and. The total mercury content was expressed as mg/kg.

### 4.5. Oxidative Biochemistry Analyses

#### 4.5.1. Antioxidant Capacity against Peroxyl Radicals (ACAPs)

This analysis was proceeded based on Amado et al. [68]. One aliquot of the homogenate was centrifuged at 14,000 rpm at 4 °C for 10 min to collect the supernatant. From the supernatant, 200 μL was incubated with a peroxyl radical generator (2,2′-azobis-2-methylpropionamidine dihydrochloride (ABAP; 4 mM; Sigma-Aldrich, San Louis, MO, USA) in triplicate to verify the oxidation of ABAP and generate fluorescence. After the reaction with ABAP, the generated fluorescence was measured on a fluorimeter (Victor X3, Perkin Elmer, Waltham, MA, USA) at a controlled temperature of 35 °C. After the first reading to determine the fluorescence value, a total volume of 10 µL of 2′,7′ H2DCF-DA (40 nM) was added to the microplate, with cyclic readings every 5 min for 60 min. High relative areas under the curve established by a second-degree polynomial curve indicate a low antioxidant capacity to neutralize peroxyl radicals. The inverse relative difference between the area with and without ABAP was considered a measure of antioxidant capacity. The results were later expressed as the percentage of control.

#### 4.5.2. Lipid Peroxidation (LPO)

For LPO, 5 μL from total homogenate was diluted with Tris-HCl buffer (1:30, *v*:*v*) followed by quantifying total protein content by Bradford’s method [69] for data normalization. The remaining lysate was centrifuged at 5600 rpm for 10 min at 4 °C, and the supernatant was collected to determine LPO [70]. A volume of 325 μL of 10.3 mM NMFI diluted in methanol (1:3, *v*:*v*) and 75 μL of methanesulfonic acid were added to 100 μL of the standard malondialdehyde (MDA) solutions or samples and heated at 45 °C for 40 min. Then, absorbances were measured at 570 nm, and the results were expressed as nmol of MDA/mg of protein and then converted to the percentage of control.

### 4.6. Proteomic Approach and Bioinformatic Analyses

A total of six samples from each group were used. A pool of two samples was performed, and the analysis was carried out in biological triplicate. The detailed protocol is available elsewhere [18,71,72]. The samples were cryofractured using a cryogenic mill and liquid nitrogen followed by the extraction of proteins with lysis buffer (urea 7 M, thiourea 2 M, diluted in ammonium bicarbonate) in constant stirring at 4 °C; then the samples were centrifuged for 30 min at 14,000 rpm at 4 °C. Subsequently, 50 µg of protein (determined by Bradford’s method) were diluted in 50 µL of ammonium bicarbonate (50 mM), and each sample 10 µL ammonium bicarbonate (50 mM) and 25 µL Rapigest (0.2%) (Waters Co., Manchester, UK) were added and incubated at 37 °C for 30 min. Subsequently, 2.5 µL of dithiothreitol (100 mM) was added and incubated at 37 °C for 60 min, followed by 2.5 µL of 300 mM iodoacetamide (BioRad, Hercules, CA, USA) addition and incubation for 30 min at room temperature and in the dark. Then, the protein digestion was preceded by adding 10 µL of trypsin (Thermo Fisher, Waltham, MA, USA) for 14 h at 37 °C. On the following day, 10 µL of 5% trifluoroacetic acid (Sigma-Aldrich, St. Louis, MO, USA) was added for 90 min at 37 °C and centrifuged at 14,000 rpm at 6 °C for 30 min. Afterwards, the supernatants were collected and purified using C18 spin columns (Thermo Fisher, Waltham, MA, USA). The samples were resuspended in 12 µL of ADH (1 pmol·µL^−1^) + 108 µL of 3% acetonitrile (Sigma-Aldrich, St. Louis, MO, USA) and 0.1% formic acid (Thermo Fisher, Waltham, MA, USA).

The reading and identification of the peptides were performed using a nanoAcquity UPLCXevo QTof MS system (Waters, Manchester, UK) and the Protein Lynx Global Server (PLGS) software, applying the Monte-Carlo algorithm and identifying the peptides by Uniprot database for *Rattus norvegicus*. Our analyses considered *p* < 0.05 for down-regulated proteins and 1 − *p* > 0.95 for up-regulated proteins. After compiling the results, we performed the bioinformatic analysis using the Cytoscape v. 3.8 software with the ClueGO plugin to determine the functional categories of proteins based on gene ontology annotations of biological processes.

### 4.7. Apoptosis and Cytotoxicity Assays

After dissociation, 100 µL of the homogenate containing the dissociated cells were added to a 96-well microplate with 100 µL of the reagent from the Caspase-Glo 3/7 Assay System, a luminescent assay to measure caspase-3/7 activities and CytoTox-Glo (Promega System, Delft, The Netherlands), which uses a luminogenic peptide substrate to measure dead cell protease activity. Readings were performed on GloMax equipment (Promega System, Delft, The Netherlands) according to the manufacturer’s recommendations. The results were expressed in the relative luminescence unit (RLU) for cytotoxicity and relative fluorescence unit (RFU) for apoptosis and then converted into a percentage of control.

### 4.8. Morphological Analysis

A set of animals was deeply anesthetized with a solution of xylazine hydrochloride (30 mg/kg) and ketamine chloride (180 mg/kg) (i.p.). Then, after total loss of paw and corneal reflexes, the animals were perfused with heparinized saline solution (0.9%) and fixed with 4% paraformaldehyde. The samples were collected and post-fixed in Bouin’s solution for six hours and then embedded in paraplast (Sigma-Aldrich, St. Louis, MO, USA). Coronal sections (7 µm) were obtained with a manual microtome and stained by hematoxylin and eosin routine staining.

The Purkinje cell density was evaluated using a grid attached to the eyepiece of the microscope, with dimensions corresponding to an area of 0.0625 mm^2^ to count the cells in three different fields from each section. The cells are displayed in a linear form, and then, after counting the cells, a proportional area was considered of grids filled with the cells. The representative photomicrographs were taken by a DS-Fi3 microscope camera attached to the Nikon Eclipse Ci H550s bright field microscope.

### 4.9. Statistical Analyses

**Figure 8:**
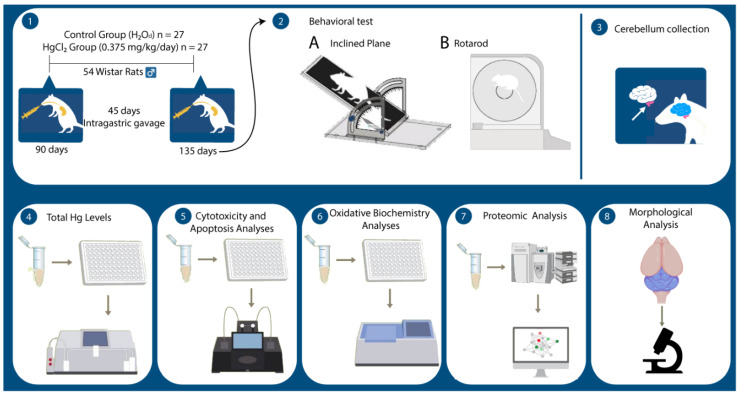
Schematic figure of all methodological steps. In (**1**), the experimental protocol of HgCl_2_ long-term exposure in adult Wistar rats, followed by motor functions assessment (**2**) by inclined plane (**A**) and rotarod (**B**) tests and then, cerebellum collection (**3**); in (**4**), quantification of total mercury (Hg) levels by atomic absorption spectrometry; in (**5**), oxidative biochemistry analyses by the determination of lipid peroxidation (LPO) levels and antioxidant activity against peroxyl radicals (ACAPs); in (**6**), proteomic analysis by mass spectrometry with bioinformatics analysis though Cytoscape software. In (**7**), evaluation of cell death nature by cytotoxicity and apoptosis assays and in (**8**), Purkinje cell counting by bright field microscopy.

After tabulation of the results obtained, the values were analyzed through the GraphPad Prism 7.0 software. The normality of data distribution was tested by the Shapiro–Wilk method, and to analyze the difference between groups, we used Student’s t-test (inclined plane score, total mercury determination, oxidative biochemistry assays, cell death nature evaluation, and morphological analysis). We also used a two-way ANOVA 2 way with Sidak’s post-test to analyze the significant difference between groups in body weight register during the experimental period and rotarod test. It was adopted *p* < 0.05 as significance in all statistical methods. For proteomic data, the statistical analysis was performed using the equipment’s software as described above. All methodological steps are summarized in Figure 8.

## 5. Conclusions

In conclusion, the results pointed out that IHg long-term exposure increases the mercury bioavailability in the cerebellum parenchyma and triggers several molecular, biochemical, and morphological impairments associated with the poor motor functions related to the cerebellum. It triggers oxidative stress and cell death by cytotoxicity and apoptosis, modulates several proteins from biological processes that may be potential new targets of researches designed to elucidate the mechanisms of damage.

## Figures and Tables

**Table 1:** Identified proteins with expression significantly altered in the cerebellum of rats of the IHg group versus control group.

^a^ Accession ID	Protein Name Description	PLGS Score	Fold Change
P34926	Microtubule-associated protein 1A	71.4	4.35
P10888	Cytochrome c oxidase subunit 4 isoform 1, mitochondrial	146.14	1.46
P0DP29	Calmodulin-1	1825.33	1.39
P0DP30	Calmodulin-2	1825.33	1.39
P0DP31	Calmodulin-3	1867.79	1.38
Q63345	Myelin-oligodendrocyte glycoprotein	290.08	1.30
P21707	Synaptotagmin-1	113.9	1.20
P07825	Synaptophysin	286.54	1.16
P68035	Actin, alpha cardiac muscle 1	10,468.68	−0.94
P39052	Dynamin-2	127.72	−0.92
P63039	60 kDa heat shock protein, mitochondrial	870.92	−0.91
P08413	Calcium/calmodulin-dependent protein kinase type II subunit beta	450.44	−0.90
P11730	Calcium/calmodulin-dependent protein kinase type II subunit gamma	323.39	−0.90
P00406	Cytochrome c oxidase subunit 2	786.54	−0.89
P0DMW0	Heat shock 70 kDa protein 1A	485.48	−0.87
Q06647	ATP synthase subunit O, mitochondrial	905.81	−0.86
P11275	Calcium/calmodulin-dependent protein kinase type II subunit alpha	220.44	−0.85
P12075	Cytochrome c oxidase subunit 5B, mitochondrial	942.79	−0.84
P15791	Calcium/calmodulin-dependent protein kinase type II subunit delta	215.3	−0.84
P60711	Actin, cytoplasmic 1	18,625.63	−0.83
P35704	Peroxiredoxin-2	2627.63	−0.82
P09951	Synapsin-1	1081.01	−0.82
P02688	Myelin basic protein	13,414.88	−0.76
P82995	Heat shock protein HSP 90-alpha	1727.77	−0.73
P07895	Superoxide dismutase [Mn], mitochondrial	514.65	−0.70
Q5U300	Ubiquitin-like modifier-activating enzyme 1	207.98	−0.69
P21575	Dynamin-1	876.32	−0.69
Q08877	Dynamin-3	202.61	−0.69
P31399	ATP synthase subunit d, mitochondrial	589.98	−0.66
P10719	ATP synthase subunit beta, mitochondrial	11,814.81	−0.65
O35244	Peroxiredoxin-6	2447.06	−0.64
P31016	Disks large homolog 4	86.82	-
Q62671	E3 ubiquitin-protein ligase UBR5	48.7	-
O88600	Heat shock 70 kDa protein 4	62.06	-
Q66HA8	Heat shock protein 105 kDa	45.92	-
Q63560	Microtubule-associated protein 6	72.62	-
P07722	Myelin-associated glycoprotein	55.31	-
P34064	Proteasome subunit alpha type-5	83.39	-
P61959	Small ubiquitin-related modifier 2	152.37	-
Q5XIF4	Small ubiquitin-related modifier 3	152.37	-
B2RYG6	Ubiquitin thioesterase OTUB1	121.2	-
Q7M767	Ubiquitin-conjugating enzyme E2 variant 2	735.2	-

+372 proteins with different status of regulation. ^a^ Accession ID according to Uniport.org database. Positive and negative values of fold change indicate up- and down-regulated proteins, respectively. Sign of—indicates exclusive expression in the control group, i.e., absent in the IHg group. Results of the comparison between the IHg group *versus* the control group.

## Data Availability

All data are available within the article and in Supplementary Materials.

## Supplementary Materials

The following are available online at https://www.mdpi.com/article/10.3390/ijms23010111/s1.

## Abbreviations

IHg	Inorganic Mercury
CNS	Central Nervous System
i.p.	Intraperitoneal
*w*/*v*	weight/volume
ABAP	2,2′-azobis-2-methylpropionamidine dihydrochloride
LPO	Lipid Peroxidation
MDA	Malondialdehyde
UPLC	Ultra performance liquid chromatography
PLGS	Protein Lynx Global SERVER
RLU	Relative Luminescence Unit
RFU	Relative Fluorescence Unit
ROS	Reactive Oxygen Species
HSP	Heat Shock Proteins
