# Supplementary material for: From Molecules to Behavior in Long-Term Inorganic Mercury Intoxication: Unraveling Proteomic Features in Cerebellar Neurodegeneration of Rats
Source: Int J Mol Sci. 2021 Dec 22;23(1):111. doi: 10.3390/ijms23010111 (PMC8745249; doi:10.3390/ijms23010111)
Supplement: Supplementary file 1 [file ijms-23-00111-s001.zip › ijms-1454469-supplementary.pdf]

Supplementary Materials

Supplementary Table S1. Identified proteins with expression significantly altered in the cerebellum of rats of group exposed to IHg (Hg) vs. control (C) group

[a]Access Number	Protein name description	PLGS Score	Fold change
P34926	Microtubule-associated protein 1A	71	4.35
P62762	Visinin-like protein 1	183	1.63
P11506	Plasma membrane calcium-transporting ATPase 2	43	1.52
P07171	Calbindin	44	1.51
P10888	Cytochrome c oxidase subunit 4 isoform 1_ mitochondrial	146	1.46
P0DP29	Calmodulin-1	1825	1.39
P0DP30	Calmodulin-2	1825	1.39
P0DP31	Calmodulin-3	1868	1.38
Q64568	Plasma membrane calcium-transporting ATPase 3	53	1.31
P04631	Protein S100-B	5719	1.30
Q812E9	Neuronal membrane glycoprotein M6-a	246	1.30
Q63345	Myelin-oligodendrocyte glycoprotein	290	1.30
P29101	Synaptotagmin-2	147	1.28
P85515	Alpha-centractin	84	1.28
P09626	Potassium-transporting ATPase alpha chain 1	172	1.27
P47861	Synaptotagmin-5	75	1.27
P80254	D-dopachrome decarboxylase	706	1.25
Q00729	Histone H2B type 1-A	806	1.23
P13638	Sodium/potassium-transporting ATPase subunit beta-2	214	1.23
P21707	Synaptotagmin-1	114	1.20
P07825	Synaptophysin	287	1.16
Q63198	Contactin-1	129	1.15
P29994	Inositol 1_4_5-trisphosphate receptor type 1	67	1.15
P0C0S7	Histone H2A.Z	1423	1.14
P12928	Pyruvate kinase PKLR	879	1.12
P06685	Sodium/potassium-transporting ATPase subunit alpha-1	839	1.11
P13233	2'_3'-cyclic-nucleotide 3'-phosphodiesterase	2858	1.09
P06687	Sodium/potassium-transporting ATPase subunit alpha-3	803	1.08
Q6Q7Y5	Guanine nucleotide-binding protein subunit alpha-13	547	1.08
Q63803	Guanine nucleotide-binding protein G(s) subunit alpha isoforms XLas	532	1.08
P63095	Guanine nucleotide-binding protein G(s) subunit alpha isoforms short	532	1.07

P06686	Sodium/potassium-transporting ATPase subunit alpha-2	753	1.06
P14659	Heat shock-related 70 kDa protein 2	1758	1.05
P02770	Serum albumin	865	0.96
Q9R1Z0	Voltage-dependent anion-selective channel protein 3	491	0.95
P68035	Actin_ alpha cardiac muscle 1	10469	0.94
P68136	Actin_ alpha skeletal muscle	10469	0.94
P63269	Actin_ gamma-enteric smooth muscle	10432	0.94
P61765	Syntaxin-binding protein 1	5526	0.94
P62738	Actin_ aortic smooth muscle	10432	0.93
P39052	Dynamin-2	128	0.92
O88989	Malate dehydrogenase_ cytoplasmic	3528	0.92
P09117	Fructose-bisphosphate aldolase C	13010	0.92
Q05962	ADP/ATP translocase 1	496	0.91
Q64559	Cytosolic acyl coenzyme A thioester hydrolase	399	0.91
P10111	Peptidyl-prolyl cis-trans isomerase A	7977	0.91
P63039	60 kDa heat shock protein_ mitochondrial	871	0.91
P04636	Malate dehydrogenase_ mitochondrial	12671	0.91
P11730	Calcium/calmodulin-dependent protein kinase type II subunit gamma	323	0.90
P61983	14-3-3 protein gamma	4079	0.90
P08413	Calcium/calmodulin-dependent protein kinase type II subunit beta	450	0.90
P62632	Elongation factor 1-alpha 2	2214	0.90
P0DMW1	Heat shock 70 kDa protein 1B	477	0.90
P07335	Creatine kinase B-type	11832	0.90
P62630	Elongation factor 1-alpha 1	2246	0.90
Q00981	Ubiquitin carboxyl-terminal hydrolase isozyme L1	733	0.89
P00406	Cytochrome c oxidase subunit 2	787	0.89
Q5RKI0	WD repeat-containing protein 1	158	0.88
P26772	10 kDa heat shock protein_ mitochondrial	1543	0.88
Q99NA5	Isocitrate dehydrogenase [NAD] subunit alpha_ mitochondrial	537	0.88
P55063	Heat shock 70 kDa protein 1-like	480	0.87
P0DMW0	Heat shock 70 kDa protein 1A	485	0.87
P49432	Pyruvate dehydrogenase E1 component subunit beta_ mitochondrial	416	0.87
Q9QWN8	Spectrin beta chain_ non-erythrocytic 2	59	0.87

Q09073	ADP/ATP translocase 2	295	0.86
Q06647	ATP synthase subunit O_ mitochondrial	906	0.86
P47858	ATP-dependent 6-phosphofructokinase_ muscle type	211	0.86
P47860	ATP-dependent 6-phosphofructokinase_ platelet type	157	0.85
P11275	Calcium/calmodulin-dependent protein kinase type II subunit alpha	220	0.85
P25809	Creatine kinase U-type_ mitochondrial	1185	0.85
P08461	Dihydrolipoyllysine-residue acetyltransferase component of pyruvate dehydrogenase complex_ mitochondrial	334	0.85
P30904	Macrophage migration inhibitory factor	3514	0.85
B0BNF1	Septin-8	589	0.85
B3GNI6	Septin-11	471	0.84
P11030	Acyl-CoA-binding protein	2961	0.84
Q68FY0	Cytochrome b-c1 complex subunit 1_ mitochondrial	575	0.84
O35179	Endophilin-A1	1190	0.84
P62804	Histone H4	5391	0.84
P25113	Phosphoglycerate mutase 1	6217	0.84
P62815	V-type proton ATPase subunit B_ brain isoform	413	0.84
P12075	Cytochrome c oxidase subunit 5B_ mitochondrial	943	0.84
Q64119	Myosin light polypeptide 6	975	0.84
P50554	4-aminobutyrate aminotransferase_ mitochondrial	532	0.84
P15791	Calcium/calmodulin-dependent protein kinase type II subunit delta	215	0.84
P47942	Dihydropyrimidinase-related protein 2	10273	0.84
Q9WVC0	Septin-7	918	0.84
Q5PQK1	Septin-10	478	0.83
P60711	Actin_ cytoplasmic 1	18626	0.83
P10860	Glutamate dehydrogenase 1_ mitochondrial	998	0.83
P11980	Pyruvate kinase PKM	5860	0.83
P05065	Fructose-bisphosphate aldolase A	4669	0.82
P35704	Peroxiredoxin-2	2628	0.82
P50399	Rab GDP dissociation inhibitor beta	1455	0.82
P09951	Synapsin-1	1081	0.82
Q05982	Nucleoside diphosphate kinase A	1313	0.81
P47728	Calretinin	424	0.81
P11442	Clathrin heavy chain 1	856	0.81

P54311	Guanine nucleotide-binding protein G(I)/G(S)/G(T) subunit beta-1	1174	0.81
P16884	Neurofilament heavy polypeptide	439	0.81
P19804	Nucleoside diphosphate kinase B	1400	0.81
P62963	Profilin-1	997	0.81
P16086	Spectrin alpha chain_ non-erythrocytic 1	376	0.81
P63259	Actin_ cytoplasmic 2	18626	0.80
P13221	Aspartate aminotransferase_ cytoplasmic	3479	0.80
Q5RKI1	Eukaryotic initiation factor 4A-II	246	0.80
P04764	Alpha-enolase	4453	0.79
P15429	Beta-enolase	1930	0.79
P00507	Aspartate aminotransferase_ mitochondrial	3666	0.79
Q5PPN4	Carbonic anhydrase-related protein	387	0.79
Q5XHZ0	Heat shock protein 75 kDa_ mitochondrial	1011	0.79
P50137	Transketolase	335	0.79
P70478	Adenomatous polyposis coli protein	32	0.78
Q8VHF5	Citrate synthase_ mitochondrial	442	0.78
A7VJC2	Heterogeneous nuclear ribonucleoproteins A2/B1	645	0.78
Q00715	Histone H2B type 1	8356	0.78
Q66HF1	NADH-ubiquinone oxidoreductase 75 kDa subunit_ mitochondrial	228	0.77
P31044	Phosphatidylethanolamine-binding protein 1	11697	0.77
P54313	Guanine nucleotide-binding protein G(I)/G(S)/G(T) subunit beta-2	765	0.76
P16290	Phosphoglycerate mutase 2	310	0.76
Q8VBU2	Protein NDRG2	1192	0.76
P46462	Transitional endoplasmic reticulum ATPase	138	0.76
Q9QUL6	Vesicle-fusing ATPase	544	0.76
P67779	Prohibitin	67	0.76
P02688	Myelin basic protein	13415	0.76
P47819	Glial fibrillary acidic protein	5630	0.75
P09606	Glutamine synthetase	1725	0.75
P42123	L-lactate dehydrogenase B chain	4364	0.75
P50398	Rab GDP dissociation inhibitor alpha	2332	0.75
Q920L2	Succinate dehydrogenase [ubiquinone] flavoprotein subunit_ mitochondrial	141	0.74
P23565	Alpha-internexin	2488	0.74
P84245	Histone H3.3	225	0.74

P07340	Sodium/potassium-transporting ATPase subunit beta-1	2934	0.74
P37805	Transgelin-3	1243	0.74
O08839	Myc box-dependent-interacting protein 1	91	0.73
P04906	Glutathione S-transferase P	413	0.73
P82995	Heat shock protein HSP 90-alpha	1728	0.73
P37377	Alpha-synuclein	1351	0.73
P07323	Gamma-enolase	7429	0.73
Q6LED0	Histone H3.1	154	0.73
P48500	Triosephosphate isomerase	9329	0.73
Q63754	Beta-synuclein	3741	0.72
P09811	Glycogen phosphorylase_ liver form	217	0.72
O35264	Platelet-activating factor acetylhydrolase IB subunit beta	175	0.72
Q63537	Synapsin-2	720	0.72
P20788	Cytochrome b-c1 complex subunit Rieske_ mitochondrial	765	0.71
Q62950	Dihydropyrimidinase-related protein 1	1559	0.71
P34058	Heat shock protein HSP 90-beta	1507	0.71
P04797	Glyceraldehyde-3-phosphate dehydrogenase	17711	0.70
P12839	Neurofilament medium polypeptide	760	0.70
P07895	Superoxide dismutase [Mn]_ mitochondrial	515	0.70
P31000	Vimentin	685	0.70
Q8K586	GTP-binding nuclear protein Ran_ testis-specific isoform	457	0.70
P45592	Cofilin-1	4087	0.70
P85834	Elongation factor Tu_ mitochondrial	158	0.70
P13668	Stathmin	820	0.70
P32551	Cytochrome b-c1 complex subunit 2_ mitochondrial	188	0.69
Q62952	Dihydropyrimidinase-related protein 3	313	0.69
P21575	Dynamin-1	876	0.69
Q08877	Dynamin-3	203	0.69
P14408	Fumarate hydratase_ mitochondrial	254	0.69
Q5U300	Ubiquitin-like modifier-activating enzyme 1	208	0.69
Q5M9I5	Cytochrome b-c1 complex subunit 6_ mitochondrial	726	0.68
P11240	Cytochrome c oxidase subunit 5A_ mitochondrial	5469	0.68
P97546	Neuroplastin	121	0.68

P16617	Phosphoglycerate kinase 1	2457	0.68
P62828	GTP-binding nuclear protein Ran	457	0.68
B5DFN2	S-adenosylhomocysteine hydrolase-like protein 1	122	0.68
Q05546	Tenascin-R	74	0.68
P84087	Complexin-2	1164	0.67
O35331	Pyridoxal kinase	225	0.67
P39069	Adenylate kinase isoenzyme 1	1151	0.66
P05708	Hexokinase-1	192	0.66
P31399	ATP synthase subunit d_ mitochondrial	590	0.66
F1LU71	AU RNA binding protein/enoyl-coenzyme A hydratase (Predicted)_ isoform CRA_a	133	0.66
P09812	Glycogen phosphorylase_ muscle form	142	0.66
P10719	ATP synthase subunit beta_ mitochondrial	11815	0.65
P63018	Heat shock cognate 71 kDa protein	6101	0.65
P19527	Neurofilament light polypeptide	2838	0.65
P63041	Complexin-1	1271	0.64
P10818	Cytochrome c oxidase subunit 6A1_ mitochondrial	1010	0.64
O35244	Peroxiredoxin-6	2447	0.64
P48675	Desmin	222	0.63
P04905	Glutathione S-transferase Mu 1	840	0.63
P61980	Heterogeneous nuclear ribonucleoprotein K	963	0.63
O88767	Protein DJ-1	3641	0.63
Q6P6V0	Glucose-6-phosphate isomerase	1843	0.61
O35077	Glycerol-3-phosphate dehydrogenase [NAD(+)]_ cytoplasmic	124	0.61
P48721	Stress-70 protein_ mitochondrial	70	0.61
P20761	Ig gamma-2B chain C region	197	0.60
Q6IG00	Keratin_ type II cytoskeletal 4	40	0.60
Q10758	Keratin_ type II cytoskeletal 8	46	0.60
Q6P6Q2	Keratin_ type II cytoskeletal 5	40	0.59
Q4FZU2	Keratin_ type II cytoskeletal 6A	41	0.59
Q6IG12	Keratin_ type II cytoskeletal 7	46	0.59
Q4V7C7	Actin-related protein 3	106	0.58
Q6IG05	Keratin_ type II cytoskeletal 75	40	0.58

Q6P9V9	Tubulin alpha-1B chain	19568	0.58
Q9R063	Peroxiredoxin-5_ mitochondrial	1574	0.58
Q5XIF6	Tubulin alpha-4A chain	17102	0.58
Q4QRB4	Tubulin beta-3 chain	17303	0.58
Q3KRE8	Tubulin beta-2B chain	20340	0.57
P02091	Hemoglobin subunit beta-1	12645	0.57
Q68FR8	Tubulin alpha-3 chain	16700	0.57
P85108	Tubulin beta-2A chain	20368	0.57
Q6P9T8	Tubulin beta-4B chain	24062	0.57
P69897	Tubulin beta-5 chain	20595	0.57
P27881	Hexokinase-2	58	0.55
P02262	Histone H2A type 1	8926	0.55
P0C169	Histone H2A type 1-C	8926	0.54
P0CC09	Histone H2A type 2-A	8926	0.54
Q4FZT6	Histone H2A type 3	8926	0.54
P27139	Carbonic anhydrase 2	275	0.54
P0C170	Histone H2A type 1-E	8926	0.54
Q64598	Histone H2A type 1-F	8926	0.54
Q00728	Histone H2A type 4	8926	0.54
A9UMV8	Histone H2A.J	8926	0.54
P11348	Dihydropteridine reductase	586	0.53
Q6AY56	Tubulin alpha-8 chain	13045	0.53
P68370	Tubulin alpha-1A chain	19965	0.53
P02564	Myosin-7	19	0.52
Q6AYZ1	Tubulin alpha-1C chain	15961	0.51
P84092	AP-2 complex subunit mu	375	0.50
Q9ESV6	Glyceraldehyde-3-phosphate dehydrogenase_ testis-specific	778	0.49
P11517	Hemoglobin subunit beta-2	3847	0.49
P21818	Stathmin-2	219	0.41
Q6URK4	Heterogeneous nuclear ribonucleoprotein A3	221	0.37
Q66HA4	Tax1-binding protein 1 homolog	50	0.36
Q5U4E6	Golgin subfamily A member 4	48	0.30

P56571	ES1 protein homolog_ mitochondrial	134	0.04
P52760	2-iminobutanoate/2-iminopropanoate deaminase	186.87	-
Q5XI79	2-oxoglutarate dehydrogenase_ mitochondrial	239.61	-
O70352	3-hydroxyacyl-CoA dehydrogenase type-2	200.64	-
P38983	40S ribosomal protein AS	214.63	-
P19945	60S acidic ribosomal protein P0	45.45	-
P19944	60S acidic ribosomal protein P1	1682.8	-
P49912	Acidic leucine-rich nuclear phosphoprotein 32 family member A	119.78	-
Q5M7U7	Actin-related protein 2	134.84	-
Q99PD5	Actin-related protein 2/3 complex subunit 1A	455.11	-
P85971	Actin-related protein 2/3 complex subunit 2	163.49	-
P10760	Adenosylhomocysteinase	33.29	-
Q9WUS0	Adenylate kinase 4_ mitochondrial	140.66	-
P52481	Adenylyl cyclase-associated protein 2	102.75	-
Q8CGZ2	Afadin- and alpha-actinin-binding protein	215.92	-
P50475	Alanine--tRNA ligase_ cytoplasmic	63.3	-
P14046	Alpha-1-inhibitor 3	41.1	-
P48037	Annexin A6	113.2	-
P52303	AP-1 complex subunit beta-1	80.86	-
P62944	AP-2 complex subunit beta	162.63	-
P04639	Apolipoprotein A-I	283.68	-
P02651	Apolipoprotein A-IV	123.7	-
Q78E60	Aryl hydrocarbon receptor nuclear translocator 2	52.16	-
Q5U318	Astrocytic phosphoprotein PEA-15	72.01	-
P35434	ATP synthase subunit delta_ mitochondrial	491.92	-
D3ZAF6	ATP synthase subunit f_ mitochondrial	137.3	-
P35435	ATP synthase subunit gamma_ mitochondrial	56.48	-
P16638	ATP-citrate synthase	44.18	-
Q5U216	ATP-dependent RNA helicase DDX39A	61.39	-
Q05764	Beta-adducin	33.03	-
P70645	Bleomycin hydrolase	126.44	-
P55068	Brevican core protein	137.32	-

Q568Z7	Calcium and integrin-binding family member 2	99.59	-
Q8K3P6	Calcium-binding mitochondrial carrier protein SCaMC-2	190.57	-
D3Z8E6	Calmodulin-regulated spectrin-associated protein 1	50.83	-
P09456	cAMP-dependent protein kinase type I-alpha regulatory subunit	73.13	-
P47727	Carbonyl reductase [NADPH] 1	121.96	-
Q9WU82	Catenin beta-1	193.95	-
P24268	Cathepsin D	166.11	-
P08081	Clathrin light chain A	152.01	-
B1H228	Coiled-coil domain-containing protein 114	34.58	-
P01026	Complement C3	28.25	-
Q9Z2F5	C-terminal-binding protein 1	129.27	-
Q9EQH5	C-terminal-binding protein 2	45.62	-
O08651	D-3-phosphoglycerate dehydrogenase	239.92	-
O35078	D-amino-acid oxidase	120.9	-
Q01205	Dihydrolipoyllysine-residue succinyltransferase component of 2-oxoglutarate dehydrogenase complex_ mitochondrial	105.38	-
Q9JHU0	Dihydropyrimidinase-related protein 5	81.23	-
Q5U2P0	DIS3-like exonuclease 1	41.29	-
Q62696	Disks large homolog 1	85.81	-
Q63622	Disks large homolog 2	78.06	-
P31016	Disks large homolog 4	86.82	-
Q9WUL0	DNA topoisomerase 1	35.52	-
Q6AYH5	Dynactin subunit 2	108.17	-
O35303	Dynamin-1-like protein	77.85	-
Q62671	E3 ubiquitin-protein ligase UBR5	48.7	-
B5DF91	ELAV-like protein 1	51.23	-
Q8CH84	ELAV-like protein 2	64.88	-
O09032	ELAV-like protein 4	55.28	-
Q9JI66	Electrogenic sodium bicarbonate cotransporter 1	40.07	-
P13803	Electron transfer flavoprotein subunit alpha_ mitochondrial	47.11	-
Q68FU3	Electron transfer flavoprotein subunit beta	139.57	-
Q68FR6	Elongation factor 1-gamma	340.47	-
P05197	Elongation factor 2	45.8	-

Q5PPJ9	Endophilin-B2	81.41	-
A0JPN4	Endoribonuclease ZC3H12A	84.28	-
P23965	Enoyl-CoA delta isomerase 1_ mitochondrial	198.89	-
Q4G061	Eukaryotic translation initiation factor 3 subunit B	37.42	-
P00884	Fructose-bisphosphate aldolase B	550.29	-
P11762	Galectin-1	130	-
P13264	Glutaminase kidney isoform_ mitochondrial	89.02	-
P08010	Glutathione S-transferase Mu 2	44.87	-
Q9Z1B2	Glutathione S-transferase Mu 5	45.56	-
Q9Z339	Glutathione S-transferase omega-1	104.99	-
P08009	Glutathione S-transferase Yb-3	220.41	-
P82471	Guanine nucleotide-binding protein G(q) subunit alpha	241.52	-
Q9JID2	Guanine nucleotide-binding protein subunit alpha-11	47.43	-
P55205	Guanylate cyclase 2G	43.34	-
O88600	Heat shock 70 kDa protein 4	62.06	-
Q66HA8	Heat shock protein 105 kDa	45.92	-
P04256	Heterogeneous nuclear ribonucleoprotein A1	127.11	-
Q9JJ54	Heterogeneous nuclear ribonucleoprotein D0	117.88	-
Q794E4	Heterogeneous nuclear ribonucleoprotein F	113.51	-
Q8VHV7	Heterogeneous nuclear ribonucleoprotein H	220.04	-
Q6AY09	Heterogeneous nuclear ribonucleoprotein H2	152.18	-
F1LQ48	Heterogeneous nuclear ribonucleoprotein L	64.57	-
Q6WRH9	Immunoglobulin superfamily member 10	32.74	-
P52296	Importin subunit beta-1	43.29	-
Q63269	Inositol 1_4_5-trisphosphate receptor type 3	40.38	-
P97697	Inositol monophosphatase 1	158.16	-
Q68FX0	Isocitrate dehydrogenase [NAD] subunit beta_ mitochondrial	497.26	-
Q6IG02	Keratin_ type II cytoskeletal 2 epidermal	40.35	-
Q6IG03	Keratin_ type II cytoskeletal 73	40.35	-
Q2PQA9	Kinesin-1 heavy chain	51.58	-
P70615	Lamin-B1	79.31	-
Q6AXZ2	Leucine-rich repeat-containing protein 46	36.81	-

P19629	L-lactate dehydrogenase C chain	63.5	-
P43244	Matrin-3	53.06	-
Q8VIG2	Meiosis regulator and mRNA stability factor 1	40.09	-
Q63560	Microtubule-associated protein 6	72.62	-
Q5XIT1	Microtubule-associated protein RP/EB family member 3	45.41	-
Q03626	Murinoglobulin-1	14.47	-
Q6IE52	Murinoglobulin-2	11.18	-
P07722	Myelin-associated glycoprotein	55.31	-
P02600	Myosin light chain 1/3_ skeletal muscle isoform	130.41	-
P16409	Myosin light chain 3	112.89	-
Q6MG60	N(G)_N(G)-dimethylarginine dimethylaminohydrolase 2	359.64	-
P19234	NADH dehydrogenase [ubiquinone] flavoprotein 2_ mitochondrial	716.41	-
Q641Y2	NADH dehydrogenase [ubiquinone] iron-sulfur protein 2_ mitochondrial	76.19	-
Q63374	Neurexin-2	70.26	-
Q63376	Neurexin-2-beta	70.26	-
P97686	Neuronal cell adhesion molecule	313.12	-
Q5FVM4	Non-POU domain-containing octamer-binding protein	231.95	-
P13084	Nucleophosmin	140.65	-
Q7TT47	Paraplegin	46.85	-
P21807	Peripherin	52.87	-
Q9WTR8	PH domain leucine-rich repeat protein phosphatase 1	46.15	-
P97573	Phosphatidylinositol 3_4_5-trisphosphate 5-phosphatase 1	44.07	-
P16446	Phosphatidylinositol transfer protein alpha isoform	181.27	-
P38652	Phosphoglucomutase-1	96.93	-
Q4G033	Piwi-like protein 4	45.39	-
Q05030	Platelet-derived growth factor receptor beta	52.22	-
P56225	POU domain_ class 5_ transcription factor 2	71.17	-
P48679	Prelamin-A/C	171.95	-
Q9EPC6	Profilin-2	727.29	-
P10960	Prosaposin	82.9	-
P34064	Proteasome subunit alpha type-5	83.39	-
P11598	Protein disulfide-isomerase A3	232.05	-

Q63081	Protein disulfide-isomerase A6	77.17	-
Q6AYT4	Protein FAM122A	110.45	-
Q9Z0W5	Protein kinase C and casein kinase substrate in neurons protein 1	158.19	-
P68403	Protein kinase C beta type	75.02	-
Q9Z250	Protein lin-7 homolog A	473.96	-
Q792I0	Protein lin-7 homolog C	447.2	-
Q4V7D2	Protein rogdi homolog	68.61	-
Q5BJL5	Protein strawberry notch homolog 1	44.44	-
Q5XIE1	Protein THEM6	58.73	-
P22062	Protein-L-isoaspartate(D-aspartate) O-methyltransferase	695.32	-
P52873	Pyruvate carboxylase_ mitochondrial	76.02	-
Q7TNY7	Rab effector MyRIP	52.28	-
Q5FWT8	REST corepressor 2	125.26	-
Q4FZU8	Rho family-interacting cell polarization regulator 1	30.74	-
Q09167	Serine/arginine-rich splicing factor 5	70.15	-
G3V6S8	Serine/arginine-rich splicing factor 6	74.9	-
P12346	Serotransferrin	59.23	-
O35412	Signal-induced proliferation-associated 1-like protein 1	36.94	-
P61959	Small ubiquitin-related modifier 2	152.37	-
Q5XIF4	Small ubiquitin-related modifier 3	152.37	-
O35049	Sphingomyelin phosphodiesterase 3	58.75	-
Q63413	Spliceosome RNA helicase Ddx39b	262.55	-
Q02563	Synaptic vesicle glycoprotein 2A	52.35	-
Q62876	Synaptogyrin-1	143.6	-
P28480	T-complex protein 1 subunit alpha	63.99	-
Q7TPB1	T-complex protein 1 subunit delta	74.8	-
Q68FQ0	T-complex protein 1 subunit epsilon	93.83	-
Q9Z0V6	Thioredoxin-dependent peroxide reductase_ mitochondrial	114.49	-
Q920J4	Thioredoxin-like protein 1	122.61	-
Q9EQS0	Transaldolase	71.24	-
P31232	Transgelin	266.59	-
Q64428	Trifunctional enzyme subunit alpha_ mitochondrial	42.85	-

Q64560	Tripeptidyl-peptidase 2	71.89	-
P04692	Tropomyosin alpha-1 chain	49.27	-
P04177	Tyrosine 3-monooxygenase	138.73	-
B2RYG6	Ubiquitin thioesterase OTUB1	121.2	-
Q7M767	Ubiquitin-conjugating enzyme E2 variant 2	735.2	-
Q4KM73	UMP-CMP kinase	313.09	-
Q9Z270	Vesicle-associated membrane protein-associated protein A	210.28	-
Q66H43	BLOC-1-related complex subunit 6	71.81	+
Q63767	Breast cancer anti-estrogen resistance protein 1	40.68	+
Q68FR2	Bridging integrator 2	41.83	+
P29147	D-beta-hydroxybutyrate dehydrogenase_ mitochondrial	61.65	+
Q6P6T4	Echinoderm microtubule-associated protein-like 2	40.53	+
Q5XI72	Eukaryotic translation initiation factor 4H	137.81	+
P30713	Glutathione S-transferase theta-2	273.54	+
P29995	Inositol 1_4_5-trisphosphate receptor type 2	20.56	+
P24062	Insulin-like growth factor 1 receptor	38.24	+
Q566E5	KDEL motif-containing protein 2	57.23	+
Q9JKW1	Mitochondrial import inner membrane translocase subunit Tim22	189.26	+
P97685	Neurofascin	29.95	+
Q03351	NT-3 growth factor receptor	62.02	+
Q66H99	Nucleolar protein 10	30.26	+
Q65Z14	Oncostatin-M-specific receptor subunit beta	59.67	+
Q62920	PDZ and LIM domain protein 5	51.2	+
P63245	Receptor of activated protein C kinase 1	63.29	+
Q64612	Receptor-type tyrosine-protein phosphatase V	24.27	+
P11507	Sarcoplasmic/endoplasmic reticulum calcium ATPase 2	55.08	+
P18596	Sarcoplasmic/endoplasmic reticulum calcium ATPase 3	26.58	+
Q63633	Solute carrier family 12 member 5	83.03	+
P97690	Structural maintenance of chromosomes protein 3	49.55	+
Q6P6S0	Synaptosomal-associated protein 47	47.98	+
Q62880	Transcription initiation factor TFIID subunit 9B	117.34	+

[a]Accession ID according to Uniport.org database. Positive and negative values of fold change indicate up- and down-regulated proteins, respectively. Sign of – and + indicate exclusive expression in the control or exposed group, respectively. Results of the comparison between the IHg group *versus* the control group.

Supplementary Table S2. The ARRIVE guideline.

The ARRIVE guidelines 2.0: author checklist

The ARRIVE Essential 10

These items are the basic minimum to include in a manuscript. Without this information, readers and reviewers cannot assess the reliability of the findings.

Item		Recommendation	Section/line number, or reason for not reporting
**Study design**	1	For each experiment, provide brief details of study design including:<br>a. The groups being compared, including control groups. If no control group has been used, the rationale should be stated.<br>b. The experimental unit (e.g. a single animal, litter, or cage of animals).	14p and Fig.8
**Sample size**	2	a. Specify the exact number of experimental units allocated to each group, and the total number in each experiment. Also indicate the total number of animals used.<br>b. Explain how the sample size was decided. Provide details of any *a priori* sample size calculation, if done.	10p and figure captions
**Inclusion and exclusion criteria**	3	a. Describe any criteria used for including and excluding animals (or experimental units) during the experiment, and data points during the analysis. Specify if these criteria were established *a priori*. If no criteria were set, state this explicitly.<br>b. For each experimental group, report any animals, experimental units or data points not included in the analysis and explain why. If there were no exclusions, state so.<br>c. For each analysis, report the exact value of *n* in each experimental group.	10p.<br>10p.<br>10p and figure captions
**Randomisation**	4	a. State whether randomisation was used to allocate experimental units to control and treatment groups. If done, provide the method used to generate the randomisation sequence.<br>b. Describe the strategy used to minimise potential confounders such as the order of treatments and measurements, or animal/cage location. If confounders were not controlled, state this explicitly.	10p.<br>10-11p.
**Blinding**	5	Describe who was aware of the group allocation at the different stages of the experiment (during the allocation, the conduct of the experiment, the outcome assessment, and the data analysis).	11p.
**Outcome measures**	6	a. Clearly define all outcome measures assessed (e.g. cell death, molecular markers, or behavioural changes).<br>b. For hypothesis-testing studies, specify the primary outcome measure, i.e. the outcome measure that was used to determine the sample size.	10-13p.<br>10-13p.
**Statistical methods**	7	a. Provide details of the statistical methods used for each analysis, including software used.<br>b. Describe any methods used to assess whether the data met the assumptions of the statistical approach, and what was done if the assumptions were not met.	13p.<br>13p.
**Experimental animals**	8	a. Provide species-appropriate details of the animals used, including species, strain and substrain, sex, age or developmental stage, and, if relevant, weight.<br>b. Provide further relevant information on the provenance of animals, health/immune status, genetic modification status, genotype, and any previous procedures.	10p.<br>10p.
**Experimental procedures**	9	For each experimental group, including controls, describe the procedures in enough detail to allow others to replicate them, including:<br>a. What was done, how it was done and what was used.<br>b. When and how often.<br>c. Where (including detail of any acclimatisation periods).<br>d. Why (provide rationale for procedures).	10-13p.
**Results**	10	For each experiment conducted, including independent replications, report:<br>a. Summary/descriptive statistics for each experimental group, with a measure of variability where applicable (e.g. mean and SD, or median and range).<br>b. If applicable, the effect size with a confidence interval.	3-7p.

The Recommended Set

These items complement the Essential 10 and add important context to the study. Reporting the items in both sets represents best practice.

Item		Recommendation	Section/line number, or reason for not reporting
**Abstract**	11	Provide an accurate summary of the research objectives, animal species, strain and sex, key methods, principal findings, and study conclusions.	1p.
**Background**	12	a. Include sufficient scientific background to understand the rationale and context for the study, and explain the experimental approach.<br>b. Explain how the animal species and model used address the scientific objectives and, where appropriate, the relevance to human biology.	1-2p.
**Objectives**	13	Clearly describe the research question, research objectives and, where appropriate, specific hypotheses being tested.	1-2p.
**Ethical statement**	14	Provide the name of the ethical review committee or equivalent that has approved the use of animals in this study, and any relevant licence or protocol numbers (if applicable). If ethical approval was not sought or granted, provide a justification.	10p.
**Housing and husbandry**	15	Provide details of housing and husbandry conditions, including any environmental enrichment.	10p.
**Animal care and monitoring**	16	a. Describe any interventions or steps taken in the experimental protocols to reduce pain, suffering and distress.<br>b. Report any expected or unexpected adverse events.<br>c. Describe the humane endpoints established for the study, the signs that were monitored and the frequency of monitoring. If the study did not have humane endpoints, state this.	10-11p.
**Interpretation/ scientific implications**	17	a. Interpret the results, taking into account the study objectives and hypotheses, current theory and other relevant studies in the literature.<br>b. Comment on the study limitations including potential sources of bias, limitations of the animal model, and imprecision associated with the results.	3-7p.
**Generalisability/ translation**	18	Comment on whether, and how, the findings of this study are likely to generalise to other species or experimental conditions, including any relevance to human biology (where appropriate).	8-10p.
**Protocol registration**	19	Provide a statement indicating whether a protocol (including the research question, key design features, and analysis plan) was prepared before the study, and if and where this protocol was registered.	10p.
**Data access**	20	Provide a statement describing if and where study data are available.	14p.
**Declaration of interests**	21	a. Declare any potential conflicts of interest, including financial and non-financial. If none exist, this should be stated.<br>b. List all funding sources (including grant identifier) and the role of the funder(s) in the design, analysis and reporting of the study.	14-15p.
